# Construction of a clinical prediction model for osteoporosis in asymptomatic elderly population based on machine learning algorithm

**DOI:** 10.3389/fmed.2025.1607734

**Published:** 2025-09-12

**Authors:** Jiaming Wang, Siyuan Zhao, Tongping Shen, Shihao Wang

**Affiliations:** ^1^School of Information Engineering, Anhui University of Chinese Medicine, Hefei, China; ^2^School of Pharmaceutical Economics and Management, Anhui University of Chinese Medicine, Hefei, China

**Keywords:** osteoporosis, elderly, machine learning, SHAP, early diagnosis, shiny

## Abstract

**Background:**

Osteoporosis is a metabolic bone disease characterized by a decrease in the amount of bone per unit volume. It is highly prevalent and has a harsh impact on patients' lives. The development of accurate predictive models for osteoporosis is beneficial in helping physicians improve the accuracy of clinical diagnosis and provide a high-quality treatment experience for older adults.

**Method:**

In this study, a robust and accurate prediction model for osteoporosis was developed and validated based on machine learning and SHAP techniques. We validated the model using ROC, calibration, and DCA curves. The data in this paper were obtained from elderly participants in several communities in Beijing from June 2021 to May 2022, including 161 (27.6%) males and 423 (72.4%) females, 248 (42.47%) with osteoporosis and 336 (57.53%) without osteoporosis.

**Results:**

Upon comparing and assessing the predictive outcomes of 135 models utilizing a combination of 10 machine learning algorithms, we found that the KNN+RF combination algorithm performs the best in terms of prediction performance. The Sensitivity, Specificity, PPV, NPV, Precision, Recall, F1, Detection Prevalence, AUC, and Brier metrics of this combined algorithm are 0.7500, 0.6634, 0.6136, 0.7614, 0.6136, 0.7200, 0.6626, 0.5000, 0.904, and 0.1601. Calibration and decision curve analyses further demonstrated the model's potential clinical utility. Ultimately, we created the Shiny web application for osteoporosis diagnosis.

**Conclusions:**

The osteoporosis prediction model is readily generalizable and can aid physicians in efficiently screening for osteoporosis in the broader older demographic. This will facilitate rapid detection and diagnosis of the disease, as well as the formulation of improved therapeutic treatment strategies for patients.

## 1 Introduction

Osteoporosis is a systemic skeletal disease, and as one of the most prevalent metabolic disorders, its pathogenesis is characterized by a decrease in the amount of bone per unit volume, which leads to fractures. Osteoporosis has, therefore, received progressively increased attention in orthopedics and endocrinology (1, 2). In recent years, the prevalence of osteoporosis has risen due to population aging and the extension of average human lifetime (3). Each year, ~75 million people worldwide have osteoporosis (4). Projections show that by 2050, Asia is expected to have the highest prevalence of osteoporosis, accounting for 50% of global osteoporotic fractures (5). It is worth pointing out that the management of osteoporosis places a heavy burden on the economy. In the United States, the estimated cost of this burden ranges between $13.7 and $20.3 billion; in the European Union, the cost is as high as €31 billion; and in the Asia-Pacific region, the figure reaches $13 billion (6–8). The condition results in diminished mobility and wellbeing, along with the potential for fragility, fractures, and mortality. This disorder adversely affects the lives of elderly individuals with osteoporosis and imposes a considerable medical and economic cost on society. Osteoporosis is frequently undetected in both the early and late phases of the illness, resulting in many patients receiving a diagnosis only after experiencing a fragility fracture (9, 10).

Confirmation of the diagnosis of osteoporosis and assessment of bone mineral density (BMD) involves a variety of technological tools, such as ultrasonography, dual-energy CT (DECT), dual-energy X-ray absorptiometry (DXA), and multichannel convolutional neural network (MCNN)-based processing of raw radiofrequency signals from quantitative ultrasound (QUS) (11–14). Among these methods, DXA has been used as the technique of choice for assessing BMD and calculating T-scores for the diagnosis of osteoporosis due to its high accuracy and wide acceptance (15, 16). According to the guidelines issued by the World Health Organization (WHO) in 1994, osteoporosis can be diagnosed in postmenopausal women and men over 50 years of age if their T-score is not higher than −2.5 standard deviations (*T* ≤ -2.5 SD) (17). However, the critical technology for DXA testing is X-rays, which leads to limitations that potentially affect multiple systems of the body and a wide range of diseases, problems that cannot be circumvented in current treatments (18–20). Given this, early osteoporosis screening, prediction, and diagnosis are particularly critical. There is an extremely urgent need to develop scientific, rational, and easy-to-use tools for early clinical screening, prediction, and diagnosis.

Shim et al. (21) used machine learning models using gradient boosting machine (GBM), support vector machine (SVM), artificial neural network (ANN), and logistic regression (LR) methods for osteoporosis risk prediction model development, and the optimal model, ANN, was derived with a model sensitivity of 0.741 under five-fold cross-validation. Lee et al. (22) used GridSearchCV or RandomizedSearchCV to measure each model's AUC, accuracy, and F1 scores using five-fold cross-validation after selecting the optimal hyper-parameter combinations for models such as LR. A prediction model for osteoporosis based on the LR algorithm was finally constructed with a model accuracy of 0.75. While these models exhibit reasonable performance, their predictive ability may be limited by the model selection and feature inclusion strategies employed. To compensate for these shortcomings, our study introduces a novel ensemble model using a combinatorial algorithm. The diagnostic logic of the model is also made more relevant to social life by innovatively incorporating social factors (e.g., education level).

The clinical data used in this study were derived from the paper “Construction and Validation of a Nomogram Clinical Prediction Model for Predicting Osteoporosis in an Asymptomatic Elderly Population in Beijing.” In the study, the researchers constructed a clinical nomogram prediction model for osteoporosis using SPSS 26.0 and R 4.0.2 software, which was designed to assist clinicians in quickly recognizing whether a patient has osteoporosis. The study results showed that the three parameters of gender, education level, and body weight have significant predictive value for the diagnosis of osteoporosis, which can assist physicians in making a rapid and effective diagnosis. This study used machine learning techniques to retest the original study by applying 10 machine learning methods and combining them to form 135 machine learning models to establish the optimal clinical prediction model. In addition, by applying the SHAP technique, we further elucidated the importance of each parameter in the model. This approach differs from the underlying nomogram model used in earlier studies in that we utilize a combination of machine learning algorithms to achieve a higher level of prediction, along with the deployment of the Shiny program to enable online disease prediction. These methodological innovations were not present in earlier studies and represent a significant improvement in prediction performance and clinical applicability.

Ultimately, this study developed a set of diagnostic tools for osteoporosis based on the Shiny platform. It aims to improve clinicians' diagnostic accuracy and alleviate the potential side effects and financial burden associated with dual-energy X-ray absorptiometry (DXA) testing.

Unlike conventional statistical analysis approaches in medicine, machine learning techniques forecast new observations by obtaining knowledge from existing information. Nevertheless, numerous sophisticated machine learning models exhibit considerable requirements for transparency and interpretability. To clarify the predictive and evaluative mechanisms behind machine learning models, explainable artificial intelligence (XAI) techniques have been utilized in clinical research. Among these, the SHAP (Shapley Additive exPlanations) method quantifies the extent and direction of variable contributions to the predicted outcomes of machine learning models, offering a visual representation of these contributions (23).

This study utilized patients' clinical markers to predict osteoporosis through a machine learning (ML) model, with the model's prognostic results clarified by SHAP technology. This study's principal findings and contributions are summarized below:

(1) A machine learning model for accurately predicting patients with osteoporosis was successfully constructed.
(2) The combined K nearest neighbor (KNN) and random forest (RF) model demonstrated excellent performance in distinguishing patients from non-patients.
(3) This study employed the SHAP method to enhance the model's interpretability by elucidating the relative significance of various factors inside the model.
(4) We developed a diagnostic application for osteoporosis based on the Shiny platform, aiming to assist clinicians in achieving a rapid and accurate diagnosis of the disease.

## 2 Materials and methods

### 2.1 Study population

The clinical characteristics data of osteoporosis patients utilized in this study were sourced from the research titled “Construction and Validation of a Nomogram Clinical Prediction Model for Predicting Osteoporosis in an Asymptomatic Elderly Population in Beijing” (24). The data were collected in a cross-sectional study. Inclusion criteria were: (1) elderly men (age >50 years) and women (menopausal; age >50 years); (2) ability to accept and undergo BMD screening; (3) completion of a questionnaire and provision of basic physical information; (4) history of residency in Beijing of more than 5 years; and (5) voluntary participation in the study and signing of an informed consent form. Exclusion criteria: (1) previous lumbar spine or hip surgery; (2) low back pain with VAS score >3 (i.e., obvious discomfort); (3) limitation of limb movement or communication disorders or mental illness; (4) history of a malignant tumor. This study was based on published retrospective datasets and employed a multi-cohort observational study design, a secondary analysis of human data. The data used was anonymized and did not contain any sensitive personal information. All subjects were adults, and informed consent was obtained from themselves or their legal guardians at the time of data collection.

All methods were carried out in accordance with relevant guidelines and regulations, and the study protocol was approved by the Biomedical Ethics Committee of West China Hospital, Sichuan University.

### 2.2 Data set indicators and measurement criteria

This study used a combination of questionnaire research and standardized assessment to collect several clinical indicators related to osteoporosis from the participants. These indicators included age, gender, physical activity participation, educational background, body height, weight, waist size, smoking history, and alcohol consumption history. Educational background was categorized as “middle school,” “high school,” and “undergraduate” in this study. Alcohol consumption history was defined as intake of more than 50 ml at least once a week for more than 1 year and either current consumption of alcohol or no abstinence from alcohol within the past 3 years. All clinical measurements were performed by experienced professionals following established standardized procedures. Participants stood barefoot, and their height was measured with a straightedge (0.1 cm precision) from the sole of the foot to the apex of the head. Body weight was assessed utilizing an electronic scale (precision 0.1 kg) in a minimally dressed indoor environment. Waist circumference was measured at the level of the umbilicus using a tape measure with an accuracy of 0.1 cm according to the World Health Organization (WHO) anthropometric guidelines. All physical measurements were conducted twice, and the average values were documented to reduce measurement mistakes.

### 2.3 Algorithm combination approach, model development and performance evaluation

In this study, the dataset was randomly divided into a training set and a test set, which accounted for 70 and 30%, respectively. 10 machine learning algorithms were used, including Elastic Network Regression (Elastic Net), Logistic Regression (LR), Classification and Regression Trees (CART), Random Forest (RF), Support Vector Machine (SVM), Bayes, k-Nearest Neighbors (KNN), Neural Networks (NN), Fisher Discriminant Analysis (FDA) and Gradient Boosting Machine (GBM), a total of 10 algorithms, were used to analyze the data of osteoporosis patients predictively.

We innovatively tested the alpha parameters of the elastic network regression algorithms individually (with alpha ranging from 0.1 to 0.9). The combination approach used in this study is a sequential combination method, where each base learner is trained and optimized separately. Then, the predictions of some models are used as new features, which are input into another model for secondary modeling, leading to model combination and construction. We combined these algorithms two-by-two to form 135 different combinations of machine learning models for training. In this study, a systematic hyperparameter optimization of multiple machine learning models was performed using R's caret framework. Prior to each round of model combination, the key hyperparameters of each base model were first tuned using the grid search (GRID SEARCH) method to enhance their performance. All models were trained using 10-fold cross-validation to ensure robustness and accuracy. For ensemble models constructed using caretEnsemble, class probability estimation was performed using bootstrap resampling (*n* = 25), and performance was assessed using the twoClassSummary metric (e.g., for the random forest component, the mtry parameter was optimized over a range of 2–4, with an optimal value of mtry = 3. For the KNN component, the optimal number of neighbors was *k* = 5.) Subsequently, we utilized Accuracy with a 95% Confidence Interval, Sensitivity, Specificity, Positive Predictive Value (PPV), Negative Predictive Value (NPV), Precision, Recall, F1 Score, Detection Prevalence, and Brier Score to comprehensively evaluate and screen the performance of the models on the test set.

In addition, we plotted the Calibration Curves and Decision Curve Analysis (DCA) curves of the top 10 models regarding Accuracy. Further, we screened the models with the best performance by comparing metrics such as Area Under the Curve (AUC). Specifically, the first batch of models with the top 10 screening accuracies is prioritized in the subsequent selection of AUC and Brier scores to balance the consistency of discrimination and calibration capabilities, especially in cases of unbalanced categories. The final selection of the best models combines criteria such as the highest AUC and Brier metrics, the highest number of metrics in each category, and DCA curves. This process aims to validate the Accuracy of the models and their potential application in clinical prediction.

### 2.4 Statistical methods

Data analysis for this study relied on R Studio (version 4.3.0) and Python (version 3.11.0). At the initial stage, this study used a univariate analysis strategy to identify seven variables of clinical relevance, which included osteoporosis status (OP), gender, education level, height, weight, waistline, and smoking and drinking habits. In this study, we have not used multicollinearity indicators (e.g., variance inflation factor VIF, correlation matrix) for validation. However, we have ensured model robustness through elastic network regularization, SHAP feature contribution analysis, and 10-fold cross-validation.

On this basis, these filtered variables were incorporated into the input parameters of 135 machine-learning models. Specifically, the Enet model uses the “glmnet” function, the LR model uses the “glm” function, the CART model uses the “rpart” function, the RF model uses the “rf” function, the SVM model uses the “svmLinear3” function, and the Bayes model uses the “bayesglm” function, the KNN model uses the “knn” function, the NN model uses the “nnet” function, the FDA model uses the “fda” function, and the GBM model uses the “gbm” function.

To evaluate the model's performance, we utilized the “plotROC,” “caret,” “autoReg,” “pROC,” and “e1071” packages of the R software to generate baseline tables and ROC curves. Meanwhile, using Python software, we plotted the SHAP values to visualize and analyze the degree of influence of the model parameters.

This study used a two-sided *P*-value of < 0.05 to judge the results' statistical significance.

### 2.5 SHAP

**Figure 1:**
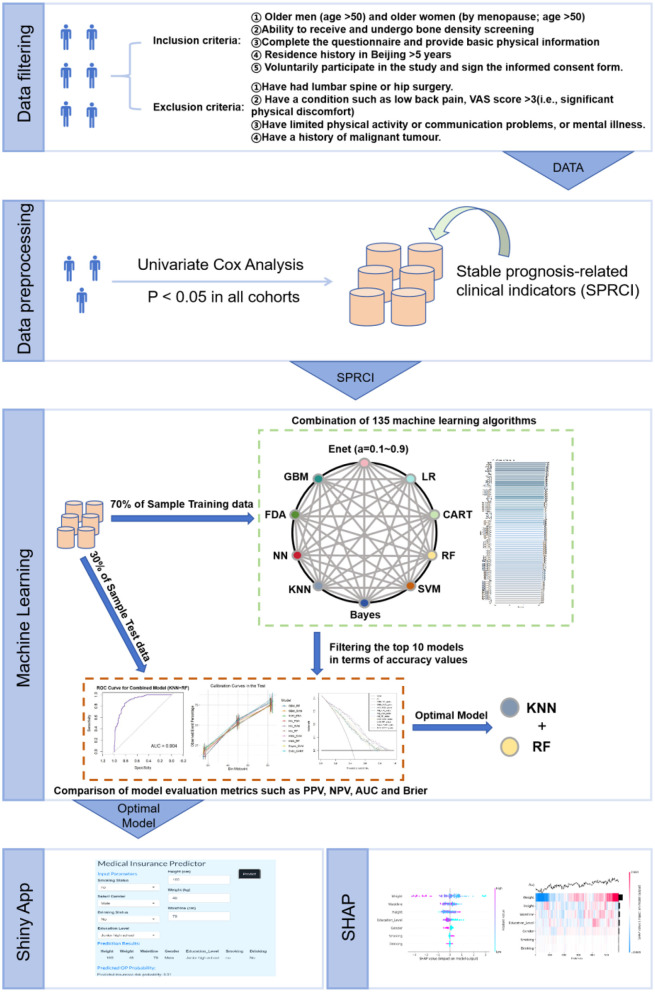
Flowchart of article techniques and methods.

This study seeks to clarify the distinct impacts of every variable that is independent in the machine learning model on the prediction outcomes, utilizing the SHAP (Shapley Additive Explanations) method. The SHAP technique is grounded in Shapley value theory, aimed at elucidating both individual and aggregate forecasts of the model. Shapley values are determined by evaluating the predicted contribution of all potential variable combinations to the observations, so assuring an equitable evaluation of each variable in the prediction. The SHAP methodology specifically seeks to elucidate the rationale for each observation's prediction by quantifying the marginal contribution of every variable that is independent to the projected result. This work delineates the approaches and methodological processes utilized, as depicted in Figure 1, which depicts the comprehensive analytical process from data preprocessing to model interpretation.

## 3 Results

### 3.1 Comparative analysis of patients' baseline characteristics table

This study includes 584 patients: 423 females and 161 males. The average age of the patients was 66.90 years, with a standard deviation of 6.45 years; the key characteristics of the patients are outlined in Table 1. No statistically significant age difference was seen between the osteoporosis (OP) group and the non-osteoporosis (N-OP) group (66.58 ± 6.74 vs. 67.34 ± 6.02 years, *P* = 0.157). However, the N-OP group had significantly higher height (163.54 ± 7.77 vs. 159.73 ± 6.89, *P* < 0.001), weight (68.19 ± 10.19 vs. 59.95 ± 7.78, *P* < 0.001) and waist circumference (86.36 ± 9.40 vs. 81.76 ± 7.76, *P* < 0.001) than the OP group.

**Table 1:** Baseline table of clinical indicators for patients with osteoporosis.

**Characteristics**	**Variables**	**No osteoporosis**	**Osteoporosis**	**All**	***P*-value**
		**(*****N*** = **336)**	**(*****N*** = **248)**	**(*****N*** = **584)**	
Gender					< 0.001
	Female	209 (62.2%)	214 (86.3%)	423 (72.4%)	
	Male	127 (37.8%)	34 (13.7%)	161 (27.6%)	
Manual_laborers					0.352
	No	225 (67%)	156 (62.9%)	381 (65.2%)	
	Yes	111 (33%)	92 (37.1%)	203 (34.8%)	
Smoking					0.008
	No	285 (84.8%)	229 (92.3%)	514 (88%)	
	Yes	51 (15.2%)	19 (7.7%)	70 (12%)	
Drinking					0.002
	No	285 (84.8%)	232 (93.5%)	517 (88.5%)	
	Yes	51 (15.2%)	16 (6.5%)	67 (11.5%)	
Education_level					< 0.001
	Junior high school	67 (19.9%)	112 (45.2%)	179 (30.7%)	
	High school	161 (47.9%)	95 (38.3%)	256 (43.8%)	
	Undergraduate	108 (32.1%)	41 (16.5%)	149 (25.5%)	
Age (years)	Mean ± SD	66.58 ± 6.74	67.34 ± 6.02	66.90 ± 6.45	0.157
Height	Mean ± SD	163.54 ± 7.77	159.73 ± 6.89	161.92 ± 7.64	< 0.001
Weight	Mean ± SD	68.19 ± 10.19	59.95 ± 7.78	64.69 ± 10.09	< 0.001
Waistline	Mean ± SD	86.36 ± 9.40	81.76 ± 7.76	84.41 ± 9.03	< 0.001

In the univariate analysis, the differences of seven factors, including Gender, Smoking, Drinking, Education Level, Height, Weight, and Waistline, were statistically significant, *P* < 0.05. Gender, a critical clinical factor, was identified in both the training and validation sets, revealing a considerably higher prevalence of osteoporosis among female patients compared to males (*P* < 0.001). Their status as manual laborers was not statistically significant (*P* > 0.05). Individuals with high school and undergraduate education were having a reduced incidence of osteoporosis relative to individuals with a middle school education (*P* < 0.001). Regarding smoking and drinking history, it was found that the proportion of smoking and drinking was relatively high in the non-OP group, a phenomenon that the gender factor may influence. After careful consideration, this study finalized the variables of OP, Gender, Smoking, Drinking, Education Level, Height, Weight, and Waistline as parameters for training and constructing 135 machine-learning models.

### 3.2 Comparative performance evaluation of machine learning models for osteoporosis detection

**Figure 2:**
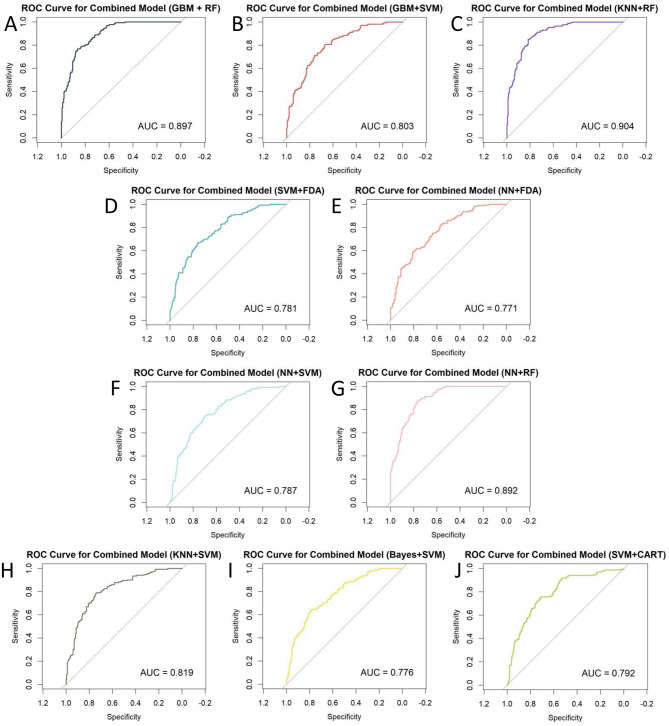
ROC curves for 10 combined machine learning models: **(A)** GBM+RF; **(B)** GBM+SVM; **(C)** KNN+RF; **(D)** SVM+FDA; **(E)** NN+FDA; **(F)** NN+SVM; **(G)** NN+RF; **(H)** KNN+SVM; **(I)** Bayes+SVM; **(J)** SVM+CART.

**Figure 3:**
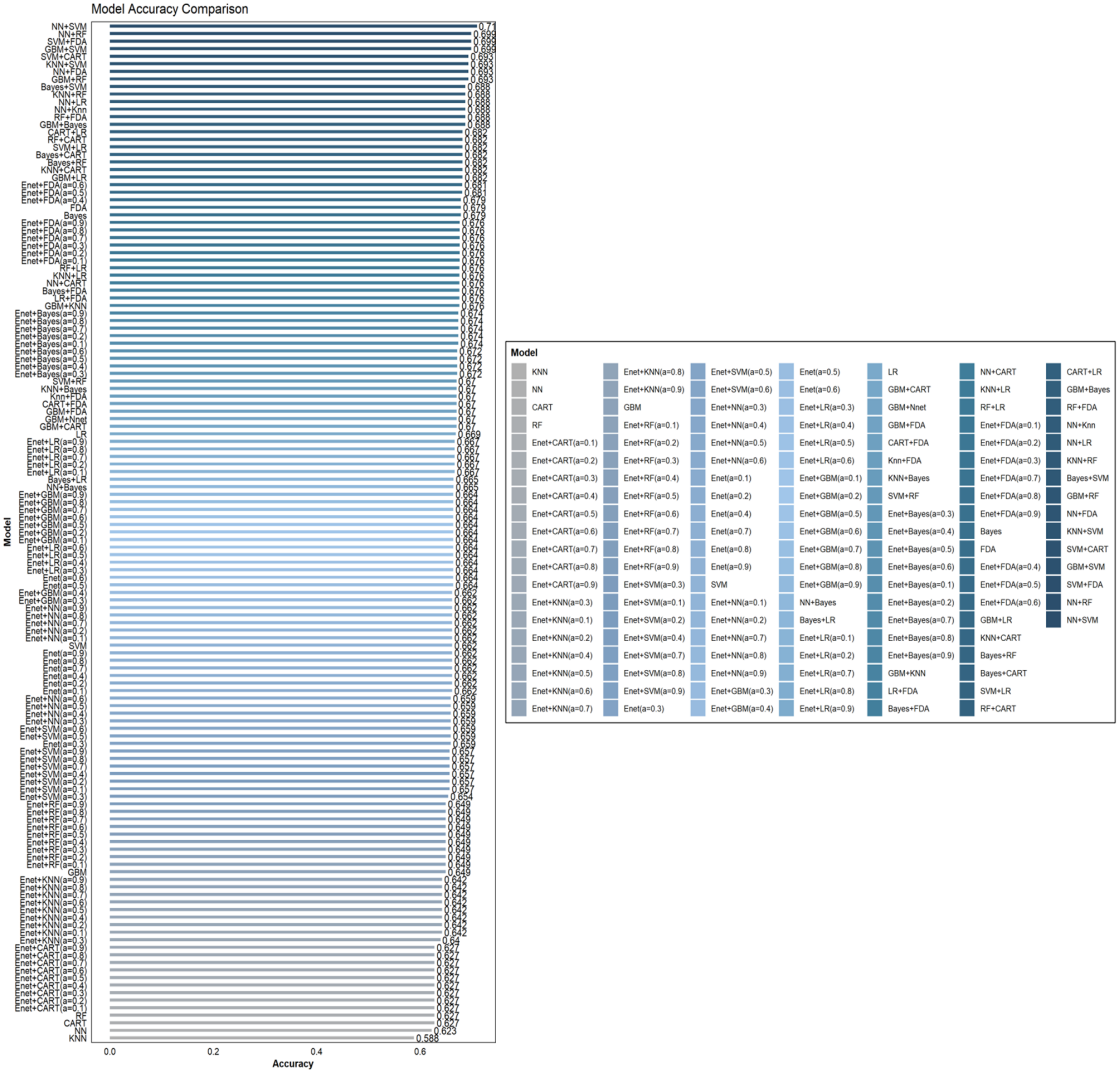
Comparison of the accuracy of 135 combined machine learning models.

This study involved the development and evaluation of 135 machine learning models on the training set, with the performance characteristics of all of them presented in Supplementary Table S1. Employing Accuracy (95% CI) as the selection criterion, we identified the 10 models with the best Accuracy and displayed their comprehensive performance statistics in Table 2. The curved AUC of these 10 models varies from 0.771 to 0.904, with the KNN+RF combo model exhibiting the highest AUC value. Figure 2 displays the associated receiver operating characteristic (ROC) curves. The NNN+SVM combo model exhibits the highest accuracy (Accuracy: 0.7102, CI: 0.6372–0.7760), whilst the accuracy of the other nine models varies between 0.6875 and 0.6989. Figure 3 illustrates the accuracy of the comparison among the 135 machine-learning models.

**Table 2:** Accuracy (95% CI) results for the top 10 machine learning models.

**Model**	**GBM+RF**	**GBM+SVM**	**SVM+FDA**	**NN+FDA**	**NN+SVM**	**NN+RF**	**KNN+SVM**	**KNN+RF**	**Bayes+SVM**	**SVM+CART**
Accuracy (95% CI)	0.6932 (0.6194, 0.7604)	0.6989 (0.6253, 0.7656)	0.6989 (0.6253, 0.7656)	0.6932 (0.6194, 0.7604)	**0.7102 (0.6372, 0.7760)**	0.6989 (0.6253, 0.7656)	0.6932 (0.6194, 0.7604)	0.6875 (0.6134, 0.7551)	0.6875 (0.6134, 0.7551)	0.6932 (0.6194, 0.7604)
Sensitivity	0.6933	0.7333	0.7467	0.7733	0.6933	0.7333	0.7200	**0.7500**	0.6133	0.7067
Specificity	0.6931	0.6733	0.6634	0.6337	0.7228	0.6733	0.6733	0.6634	**0.7426**	0.6832
PPV	0.6265	0.6250	0.6222	0.6105	**0.6500**	0.6250	0.6207	0.6136	0.6389	0.6235
NPV	0.7527	0.7727	0.7791	0.7901	0.7604	0.7727	0.7640	0.7614	0.7212	0.7582
Precision	0.6265	0.6250	0.6222	0.6105	**0.6500**	0.6250	0.6207	0.6136	0.6389	0.6235
Recall	0.6933	0.7333	**0.7467**	0.7733	0.6933	0.7333	0.7200	0.7200	0.6133	0.7067
F1	0.6582	0.6748	0.6788	**0.6824**	0.6710	0.6748	0.6667	0.6626	0.6259	0.6625
Detection prevalence	0.4716	**0.5000**	0.4114	0.4998	0.4545	**0.5000**	0.4943	**0.5000**	0.4091	0.4830
AUC	0.897	0.803	0.781	0.771	0.787	0.892	0.819	**0.904**	0.776	0.792
Brier	0.1612	0.1918	0.1931	0.1921	0.1937	0.1613	0.1887	**0.1601**	0.1940	0.1987

Individual maximum values are shown in bold.

We conducted a multidimensional comparison of the predictive efficacy of these 10 machine-learning combination algorithms. The results show that among all the combination algorithms, the KNN+RF combination model performs the best in Sensitivity and Detection Prevalence. In contrast, the NN+SVM combination model dominates Accuracy, PPV, and Precision.

In addition, nine other machine learning algorithms showed good predictive ability. To further assess the Accuracy of the models, we calculated the Brier score (brier score). In the reliability assessment of the brier score, the KNN+RF combination model outperforms the GBM+RF, GBM+SVM, SVM+FDA, NN+FDA, NN+SVM, NN+RF, KNN+SVM, Bayes+SVM, and SVM+CART combination models.

Considering each model's prediction performance, we found that the combined KNN+RF model has the optimal classification effect and robustness in recognizing OP while maintaining a high level of Accuracy. Moreover, to highlight that the superior performance of the combined KNN+RF model stems from the advantage of model integration rather than a single algorithm, this study compares it with the KNN and RF models. As shown in Supplementary Figure S2, KNN+RF outperforms both models in all evaluation metrics (including AUC, sensitivity, NPV, and F1 score, etc.), which fully reflects the advantages of the combined model in terms of discriminative power and overall classification performance. Based on this, we decided to use the combined KNN and RF model for the subsequent analysis work.

### 3.3 ML model calibration curve and DCA curve

**Figure 4:**
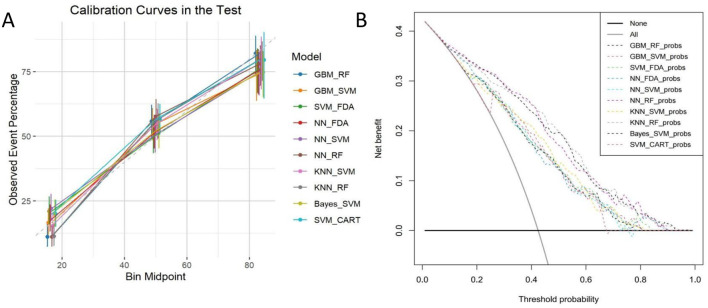
**(A)** Calibration curves; **(B)** DCA curves.

This study further validated the clinical applicability of the proposed model via calibration curve and decision curve analysis (DCA). The analysis of the calibration curve seeks to evaluate the precision of the model's predictive outcomes; a curve that closely aligns with the central diagonal indicates superior model performance. Figure 4A illustrates that the KNN+RF combination model surpassed the other nine machine-learning combination models on the calibration curve, indicating superior prediction accuracy.

Traditional evaluation of machine learning models usually focuses on diagnostic accuracy, ignoring the actual utility of the model in clinical applications. The decision curve analysis (DCA) technique bridges this gap by incorporating the preference factors of patients or clinical decision-makers to provide a comprehensive evaluation of the clinical benefits of the models and a visual presentation of the value of the models for application in the clinical setting. As shown in Figure 4B, of the 10 machine learning combination models examined, all demonstrated some clinical value, with the KNN+RF, SVM+CART, and NN+RF combination models performing most prominently in terms of clinical benefit.

### 3.4 Model interpretation and individual assessment

**Figure 5:**
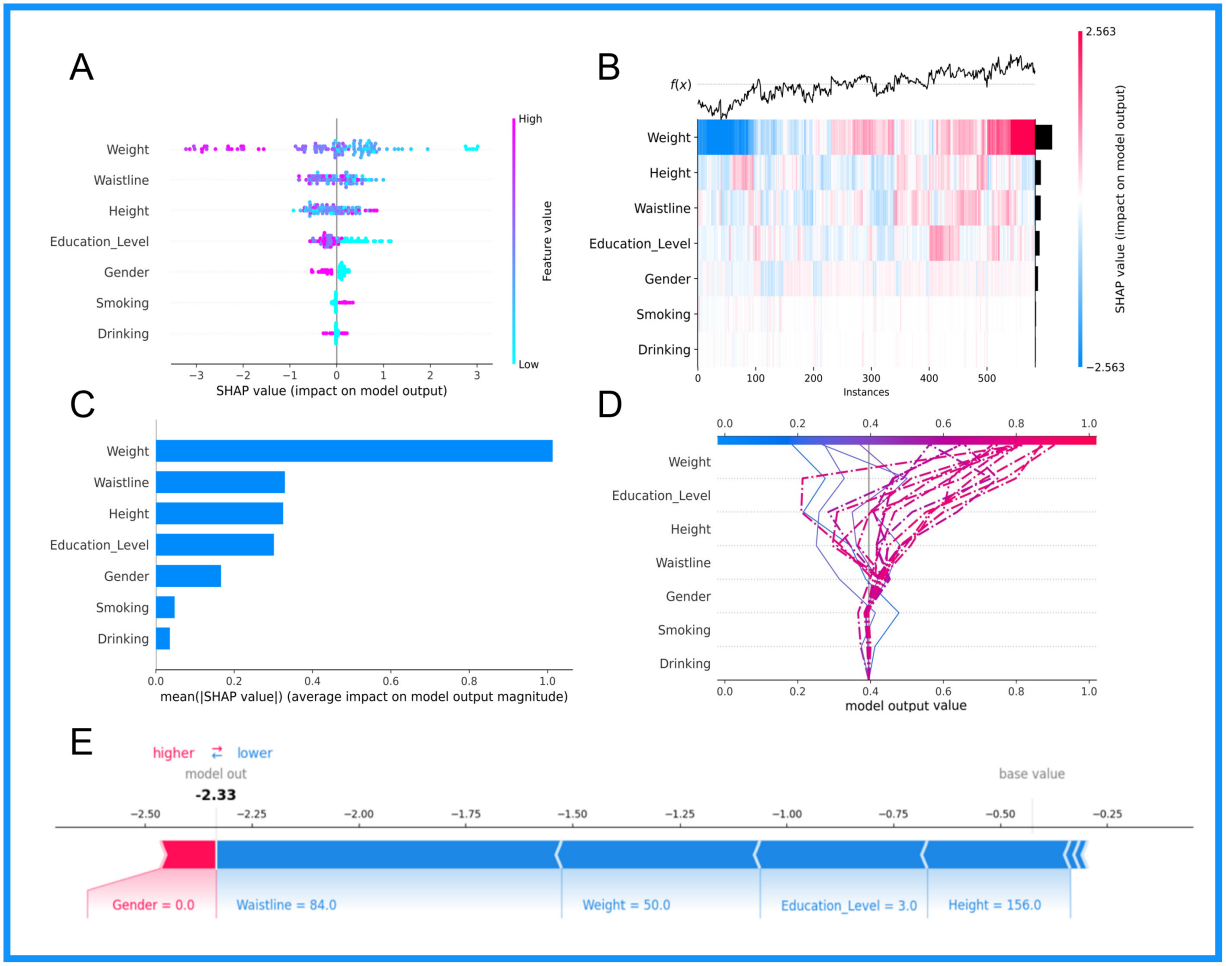
Visual representation of the combined KNN+RF model based on SHAP analysis technique: **(A)** Summary plot; **(B)** Cross-instance SHAP value heatmap; **(C)** Bar chart ranking features based on their average impact; **(D)** Dependence plot for the feature weight; **(E)** Decision plot.

This research employed the SHAP methodology to illustrate the influence of designated clinical attributes on OP within the KNN+RF model. As shown in Figures 5A–D, this study performed an interpretability analysis of global patient diagnostic indicators. Figure 5A illustrates the seven primary predictors of osteoporosis. Including Weight, Waistline, Height, Education Level, Gender, Smoking, and Drinking; the mean significance of these variables is shown in Figure 5C. Figure 5B shows the overall substructure of the dataset of patients with osteoporosis by supervised clustering, as well as the hierarchical cluster-based and explanatory similarity to rank the predictors for older participants, and the bars on the right side of the figure show the global significance of each input factor. The decision logic, base values, and predictor parameters of the model are shown in Figure 5D.

Figure 5E presents a SHAP plot for an elderly participant (non-osteoporotic patient) to demonstrate the model's interpretability. The elder participant had raised waistline, height, weight, and education levels, and the model forecasted a diminished risk of osteoporosis for this individual.

## 4 Discussion

The insidious nature of osteoporosis poses a challenge for early intervention, especially in the elderly patient population, where traditional DXA testing methods are not only invasive but also costly due to their specific physiological and physical conditions, which limits widespread screening for the disease in developing countries and regions. Therefore, how to effectively predict whether an elderly patient has osteoporosis quickly and accurately at an early stage in order to guide clinical personalized treatment has been a significant focus and difficulty in medical research.

The rise of quality and personalized medicine has placed artificial intelligence-driven prediction models in the vanguard of clinical research. This study employed a retrospective analysis to gather health data on senior participants and assess the prevalence of osteoporosis among them. We developed and validated a clinical prediction model for osteoporosis in asymptomatic individuals using machine learning techniques. The therapeutic efficacy of the created model was rigorously evaluated using contrast analysis and internal confirmation, aiming to enhance early detection and personalized treatment for elderly osteoporosis patients, hence assisting physicians in making more informed decisions. And compared to the traditional multicollinearity test, this study used the elastic network regularization and SHAP techniques. Elastic network regularization effectively reduces the effect of covariance by applying penalties to redundant features, while the SHAP technique evaluates the importance of features and filters out key predictors, thus reducing the risk of multicollinearity. In addition, 10-fold cross-validation is used to ensure model robustness and prevent overfitting.

This research employed 10 machine learning techniques to develop 135 predictive models. The integrated machine learning methods of GBM+RF, GBM+SVM, SVM+FDA, NN+FDA, NN+SVM, NN+RF, KNN+SVM, KNN+RF, Bayes+SVM, and SVM+CART shown strong performance in diagnosing osteoporosis patients. Through a thorough evaluation of the predictive efficacy of various models, we determined that the KNN+RF model exhibits superior classification performance and robustness in detecting osteoporosis (OP), achieving the highest AUC value of 90.4%. Other recent studies have proposed relevant prediction models; for example, Jang et al. (25) developed a DNN-based deep learning model using imaging and clinical data with an AUC of 0.867. Similarly, Carvalho et al. employed a comprehensive machine learning model incorporating a large number of biochemical metrics, achieving an AUC of 0.94 (26). In contrast, our model utilizes only non-invasive features and achieves an AUC of 0.904, highlighting the practical efficiency and robustness of our model, which does not rely on imaging or biochemical data. This indicates enhanced predictive capability regarding the presence or absence of osteoporosis, leading us to select the KNN+RF model for further analysis. The calibration plots indicated that the predicted curves of the combined KNN+RF model corresponded with the observed curves. DCA plots indicated that employing KNN+RF, SVM+CART, and the integrated NN+RF model for the diagnosis and prediction of OP, along with suitable therapeutic actions, is advantageous for patients in clinical practice. The impact of the identified characteristics in the KNN+RF model on OP was elucidated using the SHAP technique, revealing that the four metrics of Weight, Waistline, Height, and Education level exerted the most substantial influence on the diagnosis of OP. This finding is consistent with previous studies (27, 28).

Relevant literature has found that for osteoporotic fractures, low body weight is one of its causative risk factors, especially for older menopausal women, and wasting is one of the main factors leading to osteoporosis (29–31). This explains the higher percentage of female patients (72.4% female) in the statistical analysis of this study. Furthermore, studies have shown that body fat distribution at different sites is positively correlated with bone density, regardless of the site (femur or lumbar spine) (32). The possible reason for this is that different types of fat (e.g., android fat and gynoid fat) are involved in the endocrine regulation of bone benefits (33). Also, low body weight may lead to a decrease in body muscle mass, which can induce sarcopenia and increase the risk of falls, injuries, and fractures, leading to the complication of osteoporosis (34), which is consistent with the results of the baseline table analysis and SHAP analysis in the present study. Moreover, the protective effect of weight gain against osteoporosis can be attributed in part to muscle-derived actin, such as irisin, which has been shown to promote osteoblast differentiation and bone formation, and is positively correlated with bone mineral density (35, 36). Additionally, adipose tissue serves as an endocrine organ, secreting hormones such as adiponectin. In several studies, adiponectin has been shown to be inversely correlated with bone mineral density and adiponectin is also inversely correlated with fat content, so that weight gain will lead to an increase in bone mineral density from the point of view of endocrine regulation, which will in turn become a protective factor against osteoporosis (37, 38). These mechanisms support the observations made in our model. Gkastaris and Zhang et al. demonstrated that obesity significantly impacts society and is strongly associated with osteoporosis. The obesity risk factor is waist circumference (WC), one of the most critical risk factors for the development of osteoporosis. Among them, waist circumference (WC) is an important indicator used to assess the accumulation of abdominal fat, which is associated with the onset of many diseases (9, 39–43). In the present study, Waistline was negatively associated with the prevalence of osteoporosis, which is consistent with the findings of Murat and Saşak (44). This phenomenon differs from conventional wisdom, but it has been suggested that there is an obesity paradox in some populations, whereby moderately overweight individuals may have a better prognosis for osteoporosis, especially in women (45).

Ono et al. (46) and Pouresmaeili et al. (47) have shown that height is an independent risk factor for elderly patients with osteoporosis and that changes in height are a common clinical manifestation in elderly patients with osteoporosis accompanied by vertebral fractures and kyphosis. In addition, the level of education is also associated with the risk of developing osteoporosis. This study showed that individuals with higher levels of education have a better economic status, are more health-conscious, and are therefore less likely to develop osteoporosis. Wang et al. (48) showed a higher prevalence of osteoporosis in older adults with lower education. Meanwhile related studies point out that this may be because less educated people tend to have poorer knowledge about osteoporosis prevention and are more likely to adopt unhealthy lifestyles, including less robust health literacy (unwillingness to take medication), poorer preventive behaviors (e.g., insufficient calcium intake, lack of time and access to physical activity, etc.), and poor diets, among other conditions. In developing countries, the prevalence of osteoporosis is significantly higher than in developed countries. This difference may reflect differences in urbanization, socioeconomic status (SES), healthcare, and health education, with scholars such as Du demonstrating that lower SES and education levels are associated with a higher risk of osteoporosis (49, 50).

**Figure 6:**
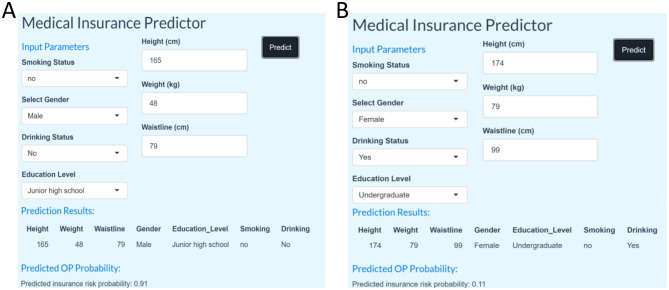
**(A)** Shiny application identified as Osteoporosis. **(B)** Shiny application identified as non-osteoporotic.

We have designed a state-of-the-art Shiny application to diagnose the presence of osteoporosis in the elderly population in order to facilitate its application and dissemination in real-world clinical practice. The application is a clinical decision support tool that provides user-friendly outputs, including personalized predictive probabilities and risk stratification, designed to help clinicians triage patients or guide follow-up testing. Clinicians can enter readily available patient data and obtain immediate predictions to support triage or follow-up recommendations. In the Shiny online prediction model, a prediction probability >0.5 is used as the threshold for clinical intervention. This reflects the default binary classification decision boundary, allowing for straightforward interpretation: patients with model-estimated probabilities >0.5 are considered to be at higher risk for osteoporosis, and further diagnostic evaluation or prophylactic treatment is recommended to clinicians. As shown in Figures 6A, B, the app is based on seven authoritative diagnostic guidelines to assist clinicians in calculating and assessing the individualized risk of developing osteoporosis. The application can be accessed via the following link: https://osteoporosispredictionmodel.shinyapps.io/medic_predict/. The shinyapps.oi server is running as shown in Supplementary Figure S1.

In clinical practice, models should be constructed and applied with holistic considerations rather than relying solely on a single feature for diagnostic prediction. Therefore, the involvement of a panel of experts is essential to assist in the diagnosis of whether a tester has osteoporosis. In addition, our findings are highly consistent with medical literature published worldwide, further validating the model's clinical relevance.

This research possesses certain limitations. First, the generalizability of the model in this study is limited by the small sample size, the fact that the data were collected from only some of the communities in Beijing, and the broad definitions of certain variables (e.g., smoking and alcohol consumption). In the future, external validation should be combined with multi-center and larger-scale data, and model calibration methods should be introduced to improve stability. Second, the model did not incorporate important clinical factors, such as comorbidities and biochemical indicators (e.g., vitamin D and serum calcium), and was based on only four anthropometric indicators, which may not fully reflect the complex etiology of osteoporosis. Third, FRAX is a risk assessment tool recommended by international guidelines (e.g., NOF, IOF) and contains clinical factors and optional BMD data (51). In contrast, the model in this study relies only on basic signs and demographic characteristics, which are suitable for resource-limited scenarios. Although not directly compared with FRAX, the predictive performance of both should be evaluated in the future, and their integration potential should be explored to enhance clinical utility. Weight, Waistline, Height, and Education level are independent predictors of osteoporosis in elderly patients. The clinical prediction model constructed in this study based on these four independent predictors can realize the accurate diagnosis of osteoporosis patients and assists physicians in devising a more evidence-based treatment plan to enhance patient prognosis and minimize societal health expenditures. Although our model predicts good results, some overfitting may occur due to issues such as data limitations. It should be used as a broad screening tool, and actual diagnosis still requires expert input and incorporation of other relevant clinical evidence. Future studies will include prospective validation using DXA as the gold standard, as well as collection of clinician feedback to assess usability, acceptance, and consistency with diagnostic outcomes. Moreover, in future studies, we will further delve into the correlations among the predictors to enhance the model's ability to identify and explain variable interactions.

## 5 Conclusion

This study used 10 machine learning methods such as Enet, LR, CART, RF, SVM, Bayes, KNN, NN, FDA, and GBM. We constructed 135 different machine learning models by combining them in order to realize the diagnosis of elderly osteoporosis patients. This research examines the efficacy of machine learning methodologies in clinical forecasting. The study's results indicate that machine learning methods perform effectively in diagnosing osteoporosis, with the combined KNN and RF model exhibiting the most superior classification efficacy and robustness, while all other model metrics also demonstrate commendable performance. Furthermore, we have created a Shiny-based online application for osteoporosis diagnosis, designed to aid clinicians in devising a more logical treatment strategy, minimizing the adverse effects linked to DXA testing technology, thus lowering healthcare expenses and enhancing patient outcomes. For future studies we will incorporate multicenter datasets to further validate the robustness and generalizability of the model.

## Data Availability

The original contributions presented in the study are included in the article/Supplementary material, further inquiries can be directed to the corresponding authors.
