# Supplementary material for: Construction of a clinical prediction model for osteoporosis in asymptomatic elderly population based on machine learning algorithm
Source: Front Med (Lausanne). 2025 Sep 12;12:1607734. doi: 10.3389/fmed.2025.1607734 (PMC12463994; doi:10.3389/fmed.2025.1607734)
Supplement: Supplementary file 1 [file Table_1.docx]

| **Model** | **Accuracy**  **（95% CI ）** | **Sensitivity** | **Specificity** | **PPV** | **NPV** | **Precision** | **Recall** | **F1** | **Detection Prevalence** |
| --- | --- | --- | --- | --- | --- | --- | --- | --- | --- |
| Enet(a=0.1) | 0.6618(0.6136, 0.7076) | 0.6243 | 0.6894 | 0.5967 | 0.7137 | 0.5967 | 0.6243 | 0.6102 | 0.4436 |
| Enet(a=0.2) | 0.6618(0.6136, 0.7076) | 0.6243 | 0.6894 | 0.5967 | 0.7137 | 0.5967 | 0.6243 | 0.6102 | 0.4436 |
| Enet(a=0.3) | 0.6593(0.6111, 0.7052) | 0.6185 | 0.6894 | 0.5944 | 0.7105 | 0.5944 | 0.6185 | 0.6062 | 0.4412 |
| Enet(a=0.4) | 0.6618(0.6136, 0.7076) | 0.6185 | 0.6936 | 0.5978 | 0.7118 | 0.5978 | 0.6185 | 0.608 | 0.4387 |
| Enet(a=0.5) | 0.6642(0.6161, 0.7099) | 0.6127 | 0.7021 | 0.6023 | 0.7112 | 0.6023 | 0.6127 | 0.6074 | 0.4314 |
| Enet(a=0.6) | 0.6642(0.6161, 0.7099) | 0.6127 | 0.7021 | 0.6023 | 0.7112 | 0.6023 | 0.6127 | 0.6074 | 0.4314 |
| Enet(a=0.7) | 0.6618(0.6136, 0.7076) | 0.6243 | 0.6894 | 0.5967 | 0.7137 | 0.5967 | 0.6243 | 0.6102 | 0.4436 |
| Enet(a=0.8) | 0.6618(0.6136, 0.7076) | 0.6243 | 0.6894 | 0.5967 | 0.7137 | 0.5967 | 0.6243 | 0.6102 | 0.4436 |
| Enet(a=0.9) | 0.6618(0.6136, 0.7076) | 0.6243 | 0.6894 | 0.5967 | 0.7137 | 0.5967 | 0.6243 | 0.6102 | 0.4436 |
| LR | 0.6691(0.6211, 0.7146) | 0.6069 | 0.7149 | 0.6105 | 0.7119 | 0.6105 | 0.6069 | 0.6087 | 0.2574 |
| CART | 0.6275(0.5785, 0.6745) | 0.7919 | 0.5064 | 0.5415 | 0.7677 | 0.5415 | 0.7919 | 0.6432 | 0.6201 |
| RF | 0.6275(0.5785, 0.6745) | 0.5954 | 0.6511 | 0.5568 | 0.6861 | 0.5568 | 0.5954 | 0.5754 | 0.4534 |
| SVM | 0.6618(0.6136, 0.7076) | 0.6127 | 0.6979 | 0.5989 | 0.7100 | 0.5989 | 0.6127 | 0.6057 | 0.4338 |
| Bayes | 0.6789(0.6312, 0.724) | 0.6012 | 0.7362 | 0.6265 | 0.7149 | 0.6265 | 0.6012 | 0.6136 | 0.4069 |
| KNN | 0.5882(0.5388, 0.6364) | 0.5145 | 0.6426 | 0.5145 | 0.6426 | 0.5145 | 0.5145 | 0.5145 | 0.4240 |
| NN | 0.6225(0.5735, 0.6698) | 0.5376 | 0.6851 | 0.5569 | 0.668 | 0.5569 | 0.5376 | 0.5471 | 0.4093 |
| FDA | 0.6789(0.6312, 0.724) | 0.5954 | 0.7404 | 0.628 | 0.7131 | 0.628 | 0.5954 | 0.6113 | 0.4020 |
| GBM | 0.6495(0.601, 0.6958) | 0.5491 | 0.7234 | 0.5938 | 0.6855 | 0.5938 | 0.5491 | 0.5706 | 0.3922 |
| GBM+LR | 0.6818(0.6075, 0.7499) | 0.6933 | 0.6733 | 0.6118 | 0.7473 | 0.6118 | 0.6933 | 0.6500 | 0.2955 |
| GBM+CART | 0.6705(0.5957, 0.7393) | 0.6667 | 0.6733 | 0.6024 | 0.7312 | 0.6024 | 0.6667 | 0.6329 | 0.4716 |
| GBM+KNN | 0.6761(0.6016, 0.7446) | 0.7067 | 0.6535 | 0.6023 | 0.7500 | 0.6023 | 0.7067 | 0.6503 | 0.5000 |
| GBM+RF | 0.6932(0.6194, 0.7604) | 0.6933 | 0.6931 | 0.6265 | 0.7527 | 0.6265 | 0.6933 | 0.6582 | 0.4716 |
| GBM+SVM | 0.6989(0.6253, 0.7656) | 0.7333 | 0.6733 | 0.6250 | 0.7727 | 0.6250 | 0.7333 | 0.6748 | 0.5000 |
| GBM+Bayes | 0.6875(0.6134, 0.7551) | 0.6933 | 0.6832 | 0.6190 | 0.7500 | 0.6190 | 0.6933 | 0.6541 | 0.4773 |
| GBM+Nnet | 0.6705(0.5957, 0.7393) | 0.6667 | 0.6733 | 0.6024 | 0.7312 | 0.6024 | 0.6667 | 0.6329 | 0.4716 |
| GBM+FDA | 0.6705(0.5957, 0.7393) | 0.6667 | 0.6733 | 0.6024 | 0.7312 | 0.6024 | 0.6667 | 0.6329 | 0.4716 |
| LR+FDA | 0.6761(0.6016, 0.7446) | 0.6933 | 0.6634 | 0.6047 | 0.7444 | 0.6047 | 0.6933 | 0.6460 | 0.4886 |
| CART+FDA | 0.6705(0.5957, 0.7393) | 0.6667 | 0.6733 | 0.6024 | 0.7312 | 0.6024 | 0.6667 | 0.6329 | 0.4716 |
| RF+FDA | 0.6875(0.6134, 0.7551) | 0.7067 | 0.6733 | 0.6163 | 0.7556 | 0.6163 | 0.7067 | 0.6584 | 0.4886 |
| SVM+FDA | 0.6989(0.6253, 0.7656) | 0.7467 | 0.6634 | 0.6222 | 0.7791 | 0.6222 | 0.7467 | 0.6788 | 0.4114 |
| Bayes+FDA | 0.6761(0.6016, 0.7446) | 0.6933 | 0.6634 | 0.6047 | 0.7444 | 0.6047 | 0.6933 | 0.6460 | 0.4886 |
| Knn+FDA | 0.6705(0.5957, 0.7393) | 0.6933 | 0.6535 | 0.5977 | 0.7416 | 0.5977 | 0.6933 | 0.6420 | 0.4943 |
| NN+FDA | 0.6932(0.6194, 0.7604) | 0.7733 | 0.6337 | 0.6105 | 0.7901 | 0.6105 | 0.7733 | 0.6824 | 0.4998 |
| NN+Knn | 0.6875(0.6134, 0.7551) | 0.7333 | 0.6535 | 0.6111 | 0.7674 | 0.6111 | 0.7333 | 0.6667 | 0.5114 |
| NN+Bayes | 0.6648(0.5899, 0.7340) | 0.5733 | 0.7327 | 0.6143 | 0.6981 | 0.6143 | 0.5733 | 0.5931 | 0.3977 |
| NN+SVM | 0.7102(0.6372, 0.7760) | 0.6933 | 0.7228 | 0.6500 | 0.7604 | 0.6500 | 0.6933 | 0.671 | 0.4545 |
| NN+RF | 0.6989(0.6253, 0.7656) | 0.7333 | 0.6733 | 0.6250 | 0.7727 | 0.6250 | 0.7333 | 0.6748 | 0.5000 |
| NN+CART | 0.6761(0.6016, 0.7446) | 0.7067 | 0.6535 | 0.6023 | 0.7500 | 0.6023 | 0.7067 | 0.6503 | 0.5000 |
| NN+LR | 0.6875(0.6134, 0.7551) | 0.6533 | 0.7129 | 0.6282 | 0.7347 | 0.6282 | 0.6533 | 0.6405 | 0.4432 |
| KNN+Bayes | 0.6705(0.5957, 0.7393) | 0.6933 | 0.6535 | 0.5977 | 0.7416 | 0.5977 | 0.6933 | 0.6420 | 0.4943 |
| KNN+SVM | 0.6932(0.6194, 0.7604) | 0.7200 | 0.6733 | 0.6207 | 0.7640 | 0.6207 | 0.7200 | 0.6667 | 0.4943 |
| KNN+RF | 0.6875(0.6134, 0.7551) | 0.7500 | 0.6634 | 0.6136 | 0.7614 | 0.6136 | 0.7200 | 0.6626 | 0.5000 |
| KNN+CART | 0.6818(0.6075, 0.7499) | 0.7333 | 0.6436 | 0.6044 | 0.7647 | 0.6044 | 0.7333 | 0.6627 | 0.5170 |
| KNN+LR | 0.6761 (0.6016, 0.7446) | 0.7067 | 0.6535 | 0.6023 | 0.7500 | 0.6023 | 0.7067 | 0.6503 | 0.5000 |
| Bayes+SVM | 0.6875(0.6134, 0.7551) | 0.6133 | 0.7426 | 0.6389 | 0.7212 | 0.6389 | 0.6133 | 0.6259 | 0.4091 |
| Bayes+RF | 0.6818(0.6075, 0.7499) | 0.6533 | 0.7030 | 0.6203 | 0.7320 | 0.6203 | 0.6533 | 0.6364 | 0.4489 |
| Bayes+CART | 0.6818(0.6075, 0.7499) | 0.6800 | 0.6832 | 0.6145 | 0.7419 | 0.6145 | 0.6800 | 0.6456 | 0.4716 |
| Bayes+LR | 0.6648(0.5899, 0.7340) | 0.5467 | 0.7525 | 0.6212 | 0.6909 | 0.6212 | 0.5467 | 0.5816 | 0.3750 |
| SVM+RF | 0.6705(0.5957, 0.7393) | 0.6267 | 0.7030 | 0.6104 | 0.7172 | 0.6104 | 0.6267 | 0.6184 | 0.4375 |
| SVM+CART | 0.6932(0.6194, 0.7604) | 0.7067 | 0.6832 | 0.6235 | 0.7582 | 0.6235 | 0.7067 | 0.6625 | 0.483 |
| SVM+LR | 0.6818(0.6075, 0.7499) | 0.6000 | 0.7426 | 0.6338 | 0.7143 | 0.6338 | 0.6000 | 0.6164 | 0.4034 |
| RF+CART | 0.6818(0.6075, 0.7499) | 0.6800 | 0.6832 | 0.6145 | 0.7419 | 0.6145 | 0.6800 | 0.6456 | 0.4716 |
| RF+LR | 0.6761(0.6016, 0.7446) | 0.6667 | 0.6832 | 0.6098 | 0.7340 | 0.6098 | 0.6667 | 0.6369 | 0.4659 |
| CART+LR | 0.6818(0.6075, 0.7499) | 0.7200 | 0.6535 | 0.6067 | 0.7586 | 0.6067 | 0.7200 | 0.6585 | 0.5057 |
| Enet+LR(a=0.1) | 0.6667(0.6186, 0.7123) | 0.7168 | 0.6298 | 0.5877 | 0.7513 | 0.5877 | 0.7168 | 0.6458 | 0.5172 |
| Enet+LR(a=0.2) | 0.6667(0.6186, 0.7123) | 0.7168 | 0.6298 | 0.5877 | 0.7513 | 0.5877 | 0.7168 | 0.6458 | 0.5172 |
| Enet+LR(a=0.3) | 0.6642(0.6161, 0.7099) | 0.7110 | 0.6298 | 0.5857 | 0.7475 | 0.5857 | 0.7110 | 0.6423 | 0.5147 |
| Enet+LR(a=0.4) | 0.6642(0.6161, 0.7099) | 0.7110 | 0.6298 | 0.5857 | 0.7475 | 0.5857 | 0.7110 | 0.6423 | 0.5147 |
| Enet+LR(a=0.5) | 0.6642(0.6161, 0.7099) | 0.7110 | 0.6298 | 0.5857 | 0.7475 | 0.5857 | 0.7110 | 0.6423 | 0.5147 |
| Enet+LR(a=0.6) | 0.6642(0.6161, 0.7099) | 0.7110 | 0.6298 | 0.5857 | 0.7475 | 0.5857 | 0.7110 | 0.6423 | 0.5147 |
| Enet+LR(a=0.7) | 0.6667(0.6186, 0.7123) | 0.7168 | 0.6298 | 0.5877 | 0.7513 | 0.5877 | 0.7168 | 0.6458 | 0.5172 |
| Enet+LR(a=0.8) | 0.6667(0.6186, 0.7123) | 0.7168 | 0.6298 | 0.5877 | 0.7513 | 0.5877 | 0.7168 | 0.6458 | 0.5172 |
| Enet+LR(a=0.9) | 0.6667(0.6186, 0.7123) | 0.7168 | 0.6298 | 0.5877 | 0.7513 | 0.5877 | 0.7168 | 0.6458 | 0.5172 |
| Enet+CART(a=0.1) | 0.6275(0.5785, 0.6745) | 0.7919 | 0.5064 | 0.5415 | 0.7677 | 0.5415 | 0.7919 | 0.6432 | 0.6201 |
| Enet+CART(a=0.2) | 0.6275(0.5785, 0.6745) | 0.7919 | 0.5064 | 0.5415 | 0.7677 | 0.5415 | 0.7919 | 0.6432 | 0.6201 |
| Enet+CART(a=0.3) | 0.6275(0.5785, 0.6745) | 0.7919 | 0.5064 | 0.5415 | 0.7677 | 0.5415 | 0.7919 | 0.6432 | 0.6201 |
| Enet+CART(a=0.4) | 0.6275(0.5785, 0.6745) | 0.7919 | 0.5064 | 0.5415 | 0.7677 | 0.5415 | 0.7919 | 0.6432 | 0.6201 |
| Enet+CART(a=0.5) | 0.6275(0.5785, 0.6745) | 0.7919 | 0.5064 | 0.5415 | 0.7677 | 0.5415 | 0.7919 | 0.6432 | 0.6201 |
| Enet+CART(a=0.6) | 0.6275(0.5785, 0.6745) | 0.7919 | 0.5064 | 0.5415 | 0.7677 | 0.5415 | 0.7919 | 0.6432 | 0.6201 |
| Enet+CART(a=0.7) | 0.6275(0.5785, 0.6745) | 0.7919 | 0.5064 | 0.5415 | 0.7677 | 0.5415 | 0.7919 | 0.6432 | 0.6201 |
| Enet+CART(a=0.8) | 0.6275(0.5785, 0.6745) | 0.7919 | 0.5064 | 0.5415 | 0.7677 | 0.5415 | 0.7919 | 0.6432 | 0.6201 |
| Enet+CART(a=0.9) | 0.6275(0.5785, 0.6745) | 0.7919 | 0.5064 | 0.5415 | 0.7677 | 0.5415 | 0.7919 | 0.6432 | 0.6201 |
| Enet+RF(a=0.1) | 0.6495(0.6010, 0.6958) | 0.7572 | 0.5702 | 0.5647 | 0.7614 | 0.5647 | 0.7572 | 0.6469 | 0.5686 |
| Enet+RF(a=0.2) | 0.6495(0.6010, 0.6958) | 0.7572 | 0.5702 | 0.5647 | 0.7614 | 0.5647 | 0.7572 | 0.6469 | 0.5686 |
| Enet+RF(a=0.3) | 0.6495(0.6010, 0.6958) | 0.7572 | 0.5702 | 0.5647 | 0.7614 | 0.5647 | 0.7572 | 0.6469 | 0.5686 |
| Enet+RF(a=0.4) | 0.6495(0.6010, 0.6958) | 0.7572 | 0.5702 | 0.5647 | 0.7614 | 0.5647 | 0.7572 | 0.6469 | 0.5686 |
| Enet+RF(a=0.5) | 0.6495(0.6010, 0.6958) | 0.7572 | 0.5702 | 0.5647 | 0.7614 | 0.5647 | 0.7572 | 0.6469 | 0.5686 |
| Enet+RF(a=0.6) | 0.6495(0.6010, 0.6958) | 0.7572 | 0.5702 | 0.5647 | 0.7614 | 0.5647 | 0.7572 | 0.6469 | 0.5686 |
| Enet+RF(a=0.7) | 0.6495(0.6010, 0.6958) | 0.7572 | 0.5702 | 0.5647 | 0.7614 | 0.5647 | 0.7572 | 0.6469 | 0.5686 |
| Enet+RF(a=0.8) | 0.6495(0.6010, 0.6958) | 0.7572 | 0.5702 | 0.5647 | 0.7614 | 0.5647 | 0.7572 | 0.6469 | 0.5686 |
| Enet+RF(a=0.9) | 0.6495(0.6010, 0.6958) | 0.7572 | 0.5702 | 0.5647 | 0.7614 | 0.5647 | 0.7572 | 0.6469 | 0.5686 |
| Enet+SVM(a=0.1) | 0.6569(0.6086, 0.7029) | 0.6532 | 0.6596 | 0.5855 | 0.7209 | 0.5855 | 0.6532 | 0.6175 | 0.4730 |
| Enet+SVM(a=0.2) | 0.6569(0.6086, 0.7029) | 0.6532 | 0.6596 | 0.5855 | 0.7209 | 0.5855 | 0.6532 | 0.6175 | 0.4730 |
| Enet+SVM(a=0.3) | 0.6544(0.606, 0.7005) | 0.6474 | 0.6596 | 0.5833 | 0.7176 | 0.5833 | 0.6474 | 0.6137 | 0.4706 |
| Enet+SVM(a=0.4) | 0.6569(0.6086, 0.7029) | 0.6474 | 0.6638 | 0.5864 | 0.7189 | 0.5864 | 0.6474 | 0.6154 | 0.4681 |
| Enet+SVM(a=0.5) | 0.6593(0.6111, 0.7052) | 0.6416 | 0.6723 | 0.5904 | 0.7182 | 0.5904 | 0.6416 | 0.6150 | 0.4608 |
| Enet+SVM(a=0.6) | 0.6593(0.6111, 0.7052) | 0.6416 | 0.6723 | 0.5904 | 0.7182 | 0.5904 | 0.6416 | 0.6150 | 0.4608 |
| Enet+SVM(a=0.7) | 0.6569(0.6086, 0.7029) | 0.6532 | 0.6596 | 0.5855 | 0.7209 | 0.5855 | 0.6532 | 0.6175 | 0.4730 |
| Enet+SVM(a=0.8) | 0.6569(0.6086, 0.7029) | 0.6532 | 0.6596 | 0.5855 | 0.7209 | 0.5855 | 0.6532 | 0.6175 | 0.4730 |
| Enet+SVM(a=0.9) | 0.6569(0.6086, 0.7029) | 0.6532 | 0.6596 | 0.5855 | 0.7209 | 0.5855 | 0.6532 | 0.6175 | 0.4730 |
| Enet+Bayes(a=0.1) | 0.6740(0.6262, 0.7193) | 0.7110 | 0.6468 | 0.5971 | 0.7525 | 0.5971 | 0.7110 | 0.6491 | 0.5049 |
| Enet+Bayes(a=0.2) | 0.6740(0.6262, 0.7193) | 0.7110 | 0.6468 | 0.5971 | 0.7525 | 0.5971 | 0.7110 | 0.6491 | 0.5049 |
| Enet+Bayes(a=0.3) | 0.6716(0.6237, 0.7170) | 0.7052 | 0.6468 | 0.5951 | 0.7488 | 0.5951 | 0.7052 | 0.6455 | 0.5025 |
| Enet+Bayes(a=0.4) | 0.6716(0.6237, 0.7170) | 0.7052 | 0.6468 | 0.5951 | 0.7488 | 0.5951 | 0.7052 | 0.6455 | 0.5025 |
| Enet+Bayes(a=0.5) | 0.6716(0.6237, 0.7170) | 0.7052 | 0.6468 | 0.5951 | 0.7488 | 0.5951 | 0.7052 | 0.6455 | 0.5025 |
| Enet+Bayes(a=0.6) | 0.6716(0.6237, 0.7170) | 0.7052 | 0.6468 | 0.5951 | 0.7488 | 0.5951 | 0.7052 | 0.6455 | 0.5025 |
| Enet+Bayes(a=0.7) | 0.6740(0.6262, 0.7193) | 0.7110 | 0.6468 | 0.5971 | 0.7525 | 0.5971 | 0.7110 | 0.6491 | 0.5049 |
| Enet+Bayes(a=0.8) | 0.6740(0.6262, 0.7193) | 0.7110 | 0.6468 | 0.5971 | 0.7525 | 0.5971 | 0.7110 | 0.6491 | 0.5049 |
| Enet+Bayes(a=0.9) | 0.6740(0.6262, 0.7193) | 0.7110 | 0.6468 | 0.5971 | 0.7525 | 0.5971 | 0.7110 | 0.6491 | 0.5049 |
| Enet+KNN(a=0.1) | 0.6422(0.5935, 0.6887) | 0.7861 | 0.5362 | 0.5551 | 0.7730 | 0.5551 | 0.7861 | 0.6507 | 0.6005 |
| Enet+KNN(a=0.2) | 0.6422(0.5935, 0.6887) | 0.7861 | 0.5362 | 0.5551 | 0.7730 | 0.5551 | 0.7861 | 0.6507 | 0.6005 |
| Enet+KNN(a=0.3) | 0.6397(0.5910, 0.6864) | 0.7803 | 0.5362 | 0.5533 | 0.7683 | 0.5533 | 0.7803 | 0.6475 | 0.5980 |
| Enet+KNN(a=0.4) | 0.6422(0.5935, 0.6887) | 0.7803 | 0.5404 | 0.5556 | 0.7697 | 0.5556 | 0.7803 | 0.6490 | 0.5956 |
| Enet+KNN(a=0.5) | 0.6422(0.5935, 0.6887) | 0.7803 | 0.5404 | 0.5556 | 0.7697 | 0.5556 | 0.7803 | 0.6490 | 0.5956 |
| Enet+KNN(a=0.6) | 0.6422(0.5935, 0.6887) | 0.7803 | 0.5404 | 0.5556 | 0.7697 | 0.5556 | 0.7803 | 0.6490 | 0.5956 |
| Enet+KNN(a=0.7) | 0.6422(0.5935, 0.6887) | 0.7861 | 0.5362 | 0.5551 | 0.7730 | 0.5551 | 0.7861 | 0.6507 | 0.6005 |
| Enet+KNN(a=0.8) | 0.6422(0.5935, 0.6887) | 0.7861 | 0.5362 | 0.5551 | 0.7730 | 0.5551 | 0.7861 | 0.6507 | 0.6005 |
| Enet+KNN(a=0.9) | 0.6422(0.5935, 0.6887) | 0.7861 | 0.5362 | 0.5551 | 0.7730 | 0.5551 | 0.7861 | 0.6507 | 0.6005 |
| Enet+NN(a=0.1) | 0.6618(0.6136, 0.7076) | 0.7225 | 0.6170 | 0.5814 | 0.7513 | 0.5814 | 0.7225 | 0.6443 | 0.5270 |
| Enet+NN(a=0.2) | 0.6618(0.6136, 0.7076) | 0.7225 | 0.6170 | 0.5814 | 0.7513 | 0.5814 | 0.7225 | 0.6443 | 0.5270 |
| Enet+NN(a=0.3) | 0.6593(0.6111, 0.7052) | 0.7168 | 0.6170 | 0.5794 | 0.7474 | 0.5794 | 0.7168 | 0.6408 | 0.5245 |
| Enet+NN(a=0.4) | 0.6593(0.6111, 0.7052) | 0.7168 | 0.6170 | 0.5794 | 0.7474 | 0.5794 | 0.7168 | 0.6408 | 0.5245 |
| Enet+NN(a=0.5) | 0.6593(0.6111, 0.7052) | 0.7168 | 0.6170 | 0.5794 | 0.7474 | 0.5794 | 0.7168 | 0.6408 | 0.5245 |
| Enet+NN(a=0.6) | 0.6593(0.6111, 0.7052) | 0.7168 | 0.6170 | 0.5794 | 0.7474 | 0.5794 | 0.7168 | 0.6408 | 0.5245 |
| Enet+NN(a=0.7) | 0.6618(0.6136, 0.7076) | 0.7225 | 0.6170 | 0.5814 | 0.7513 | 0.5814 | 0.7225 | 0.6443 | 0.5270 |
| Enet+NN(a=0.8) | 0.6618(0.6136, 0.7076) | 0.7225 | 0.6170 | 0.5814 | 0.7513 | 0.5814 | 0.7225 | 0.6443 | 0.5270 |
| Enet+NN(a=0.9) | 0.6618(0.6136, 0.7076) | 0.7225 | 0.6170 | 0.5814 | 0.7513 | 0.5814 | 0.7225 | 0.6443 | 0.5270 |
| Enet+FDA(a=0.1) | 0.6765(0.6287, 0.7217) | 0.6879 | 0.6681 | 0.6041 | 0.7441 | 0.6041 | 0.6879 | 0.6432 | 0.4828 |
| Enet+FDA(a=0.2) | 0.6765(0.6287, 0.7217) | 0.6879 | 0.6681 | 0.6041 | 0.7441 | 0.6041 | 0.6879 | 0.6432 | 0.4828 |
| Enet+FDA(a=0.3) | 0.6765(0.6287, 0.7217) | 0.6879 | 0.6681 | 0.6041 | 0.7441 | 0.6041 | 0.6879 | 0.6432 | 0.4828 |
| Enet+FDA(a=0.4) | 0.6789(0.6312, 0.724) | 0.6879 | 0.6723 | 0.6071 | 0.7453 | 0.6071 | 0.6879 | 0.6450 | 0.4804 |
| Enet+FDA(a=0.5) | 0.6814(0.6337, 0.7264) | 0.6879 | 0.6766 | 0.6103 | 0.7465 | 0.6103 | 0.6879 | 0.6467 | 0.4779 |
| Enet+FDA(a=0.6) | 0.6814(0.6337, 0.7264) | 0.6879 | 0.6766 | 0.6103 | 0.7465 | 0.6103 | 0.6879 | 0.6467 | 0.4779 |
| Enet+FDA(a=0.7) | 0.6765(0.6287, 0.7217) | 0.6879 | 0.6681 | 0.6041 | 0.7441 | 0.6041 | 0.6879 | 0.6432 | 0.4828 |
| Enet+FDA(a=0.8) | 0.6765(0.6287, 0.7217) | 0.6879 | 0.6681 | 0.6041 | 0.7441 | 0.6041 | 0.6879 | 0.6432 | 0.4828 |
| Enet+FDA(a=0.9) | 0.6765(0.6287, 0.7217) | 0.6879 | 0.6681 | 0.6041 | 0.7441 | 0.6041 | 0.6879 | 0.6432 | 0.4828 |
| Enet+GBM(a=0.1) | 0.6642(0.6161, 0.7099) | 0.6936 | 0.6426 | 0.5882 | 0.7402 | 0.5882 | 0.6936 | 0.6366 | 0.5000 |
| Enet+GBM(a=0.2) | 0.6642(0.6161, 0.7099) | 0.6936 | 0.6426 | 0.5882 | 0.7402 | 0.5882 | 0.6936 | 0.6366 | 0.5000 |
| Enet+GBM(a=0.3) | 0.6618(0.6136, 0.7076) | 0.6879 | 0.6426 | 0.5862 | 0.7366 | 0.5862 | 0.6879 | 0.6330 | 0.4975 |
| Enet+GBM(a=0.4) | 0.6618(0.6136, 0.7076) | 0.6879 | 0.6426 | 0.5862 | 0.7366 | 0.5862 | 0.6879 | 0.6330 | 0.4975 |
| Enet+GBM(a=0.5) | 0.6642(0.6161, 0.7099) | 0.6821 | 0.6511 | 0.5900 | 0.7356 | 0.5900 | 0.6821 | 0.6327 | 0.4902 |
| Enet+GBM(a=0.6) | 0.6642(0.6161, 0.7099) | 0.6821 | 0.6511 | 0.5900 | 0.7356 | 0.5900 | 0.6821 | 0.6327 | 0.4902 |
| Enet+GBM(a=0.7) | 0.6642(0.6161, 0.7099) | 0.6936 | 0.6426 | 0.5882 | 0.7402 | 0.5882 | 0.6936 | 0.6366 | 0.5000 |
| Enet+GBM(a=0.8) | 0.6642(0.6161, 0.7099) | 0.6936 | 0.6426 | 0.5882 | 0.7402 | 0.5882 | 0.6936 | 0.6366 | 0.5000 |
| Enet+GBM(a=0.9) | 0.6642(0.6161, 0.7099) | 0.6936 | 0.6426 | 0.5882 | 0.7402 | 0.5882 | 0.6936 | 0.6366 | 0.5000 |
