# Supplementary figures and images for: Construction of a clinical prediction model for osteoporosis in asymptomatic elderly population based on machine learning algorithm
Source: Front Med (Lausanne). 2025 Sep 12;12:1607734. doi: 10.3389/fmed.2025.1607734 (PMC12463994; doi:10.3389/fmed.2025.1607734)

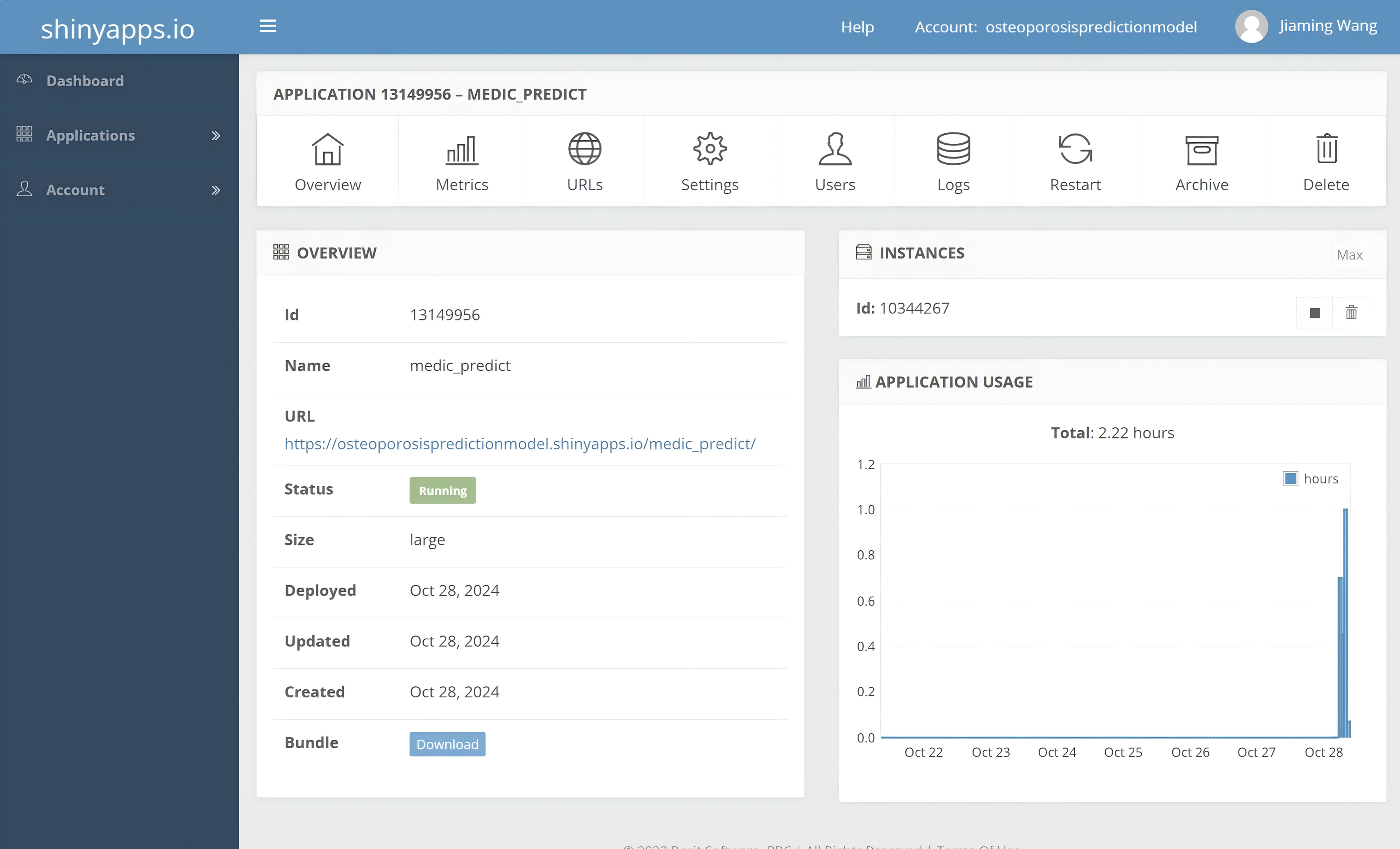

Supplement: Supplementary file 2 [file Image_1.tif]

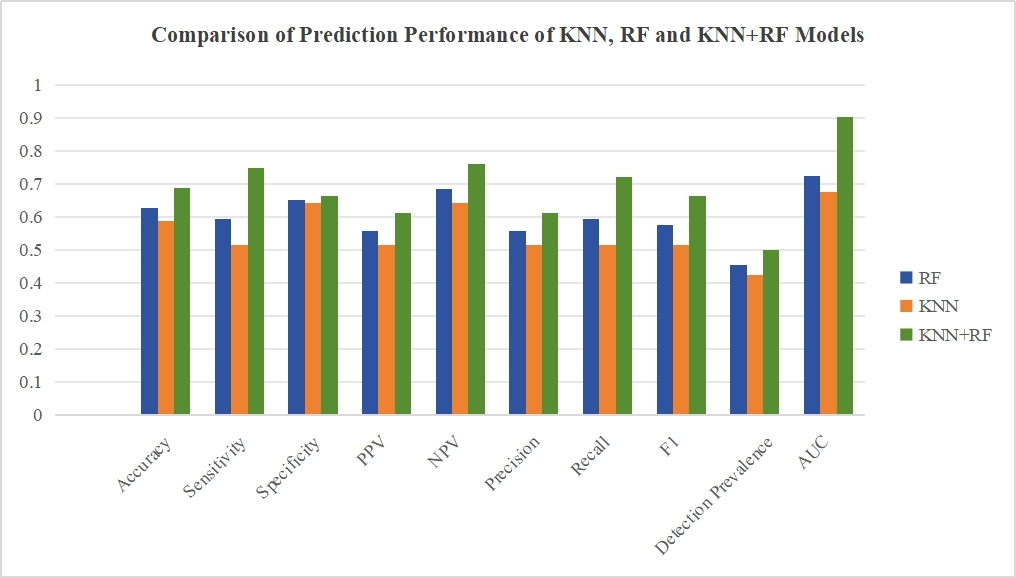

Supplement: Supplementary file 3 [file Image_2.tif]
